# Vitamin C improves microvascular reactivity and peripheral tissue perfusion in septic shock patients

**DOI:** 10.1186/s13054-022-03891-8

**Published:** 2022-01-21

**Authors:** Jean-Rémi Lavillegrand, Lisa Raia, Tomas Urbina, Geoffroy Hariri, Paul Gabarre, Vincent Bonny, Naïke Bigé, Jean-Luc Baudel, Arnaud Bruneel, Thierry Dupre, Bertrand Guidet, Eric Maury, Hafid Ait-Oufella

**Affiliations:** 1 Assistance Publique - Hôpitaux de Paris (AP-HP), Hôpital Saint-Antoine, Service de réanimation médicale, 184 rue du Faubourg Saint-Antoine, 75571 Paris Cedex 12, France; 2 Sorbonne Université, Paris, France; 3 Laboratoire de Biochimie, Hôpital Bichat, Université de Paris, Paris, France; 4 Inserm U1136, Paris, France; 5 Centre de Recherche Cardiovasculaire de Paris (PARCC), Paris, France

**Keywords:** Sepsis, Vitamin C, Mottling, Tissue perfusion, Microvascular function

## Abstract

**Background:**

Vitamin C has potential protective effects through antioxidant and anti-inflammatory properties. However, the effect of vitamin C supplementation on microvascular function and peripheral tissue perfusion in human sepsis remains unknown. We aimed to determine vitamin C effect on microvascular endothelial dysfunction and peripheral tissue perfusion in septic shock patients.

**Methods:**

Patients with septic shock were prospectively included after initial resuscitation. Bedside peripheral tissue perfusion and skin microvascular reactivity in response to acetylcholine iontophoresis in the forearm area were measured before and 1 h after intravenous vitamin C supplementation (40 mg/kg). Norepinephrine dose was not modified during the studied period.

**Results:**

We included 30 patients with septic shock. SOFA score was 11 [8–14], SAPS II was 66 [54–79], and in-hospital mortality was 33%. Half of these patients had vitamin C deficiency at inclusion. Vitamin C supplementation strongly improved microvascular reactivity (AUC 2263 [430–4246] vs 5362 [1744–10585] UI, *p* = 0.0004). In addition, vitamin C supplementation improved mottling score (*p* = 0.06), finger-tip (*p* = 0.0003) and knee capillary refill time (3.7 [2.6–5.5] vs 2.9 [1.9–4.7] s, *p* < 0.0001), as well as and central-to-periphery temperature gradient (6.1 [4.9–7.4] vs 4.6 [3.4–7.0] °C, *p* < 0.0001). The beneficial effects of vitamin C were observed both in patients with or without vitamin C deficiency.

**Conclusion:**

In septic shock patients being resuscitated, vitamin C supplementation improved peripheral tissue perfusion and microvascular reactivity whatever plasma levels of vitamin C.

ClinicalTrials.gov Identifier: NCT04778605 registered 26 January 2021.

**Supplementary Information:**

The online version contains supplementary material available at 10.1186/s13054-022-03891-8.

## Introduction

Sepsis is a common and life-threatening condition that develops in response to bacterial injury. Around 50 millions of incident cases of sepsis are recorded worldwide every year. In the USA, around 535 cases of sepsis occur annually per 100,000 people, accounting for more than USD 23 billion in annual US hospital expenditures [1]. Despite improvement in early resuscitation, sepsis-related disability and mortality remain unacceptably high [2]. Therefore, in association with symptomatic correction of acute circulatory failure and infection source control, there is urgent need for novel therapies to limit sepsis-induced tissue damage and organ failure.

Sepsis pathophysiology is complex, with immune response dysregulation, coagulation activation and oxidative burst affecting cardiac and endothelial cell function, resulting in impaired microvascular blood flow*,* tissue hypoperfusion and ultimately life-threatening organ failure [3]. Several studies have reported that the severity [4] and persistence [5] of microvascular blood flow alterations are closely correlated with patient prognosis. At bedside, impaired peripheral tissue perfusion evaluated using mottling score [6], capillary refill time [7] or temperature gradient [8] has been associated with poor outcome.

Recently, vitamin C supplementation (Ascorbic acid) has been proposed as a potential “pleiotropic” form of therapy, interacting with multiple pathologic pathways in sepsis. Several potential beneficial effects of vitamin C have been reported in both animal [9, 10] and human studies [11], including (1) antioxidant properties (scavenging of reactive oxygen species) [12, 13], (2) downregulation of pro-inflammatory gene expression (cytokines, chemokines), (3) restoration of immune cell activity [14–16], (4) downregulation of coagulation gene expression [17]. Experimental studies have also reported that vitamin C could modulate endothelial function [18, 19]. However, the in vivo effect of vitamin C on microvascular blood flow and tissue perfusion in sepsis patients with severe infections has never been investigated before.

In this study, we aimed to prospectively explore the effects of vitamin C supplementation on both endothelial-dependent microvascular reactivity and bedside peripheral tissue perfusion in septic shock patients.

## Materiel and methods

### Included patients

We conducted a prospective study in an 18-bed intensive care unit (ICU) in a tertiary teaching hospital. During a 6-month period, patients older than 18 years admitted for septic shock were included. Septic shock was defined according to the Third International Consensus Definitions for Sepsis and Septic Shock [20]. We included resuscitated patients within the first 24 h of vasopressor initiation. Exclusion criteria were pregnancy, forearm skin lesions, important soft tissue edema and agitation.

After initial therapeutic management, including antibiotic administration, fluid infusion (30 mL/kg) and norepinephrine infusion to maintain a MAP > 65 mmHg, as well as infection focus control when available, patients received intravenous (IV) vitamin C (40 mg/kg) over 30 min [21]. We compared global hemodynamic and tissue perfusion parameters before and 1 h after vitamin C supplementation, as well as skin microvascular endothelial reactivity (see below). No intervention was done during microvascular exploration (Unchanged vasopressor dose, no injection of fluid, steroid or inodilator).

### Assessment of skin microcirculation reactivity

The skin microvascular reactivity was measured in the forearm area by transdermal iontophoresis of acetylcholine (Ach) [22]. This non-invasive technique allows local transfer of Ach across the skin, which produces vasomotor action on subcutaneous capillaries [23, 24]. Ach solution and a weak electrical current are applied onto the skin, creating local differences in electrical potential and active migration of ions and molecules bearing a net electrical charge through epithelial layers. The direction and speed of migration can be adjusted using polarity and the current’s magnitude. The total amount of Ach delivered into the skin is related to the current and duration of application (i.e., electrical charge). Acetylcholine acts as an endothelium-dependent vasodilator [25], which induces the production of nitric oxide (NO) after stimulation of the endothelial NO-Synthase. Next, NO induces smooth muscle cells relaxation by activating guanylate cyclase that is responsible for vasodilation and increased blood flow.

The iontophoresis drug delivery chamber was attached to the flexor surface of the non-dominant forearm. The negative lead of the current source was attached to the drug delivery chamber, and the positive lead (i.e., reference electrode) to a conductive hydrogel pad fixed onto the wrist. After measurement of baseline blood flow for 60 s, three successive applications of Ach were made, every 60 s, using anodal current (0.12 mA for 12 s each). The drug delivery chamber was loaded with 80 μL of Ach (Miochol®). Variations of blood flow in the skin were assessed by Laser-Doppler Flowmetry technique. A Laser-Doppler Flowmeter probe (Periflux 5000, Perimed), embedded within a heating drug delivery chamber, was used in combination with a current-controlled delivery device (PeriIont, Perimed). Laser-Doppler Flowmeter signals were recorded continuously using an interfaced computer with acquisition software (Perisoft, Perimed).

Baseline blood flow (BF) and area-under-the-curve (AUC) of BF recorded during a standardized 10-min period were recorded (Additional file 1). Skin blood flow monitoring and analysis were performed by an independent physician who did not participate in patient care.

### Data collection

Patients’ characteristics were prospectively collected: age, sex, previous chronic illness, severity of illness evaluated by the Sequential Organ Failure Assessment score (SOFA score) at inclusion [26], source of sepsis, mode of mechanical ventilation, and vasopressor dose. Biological parameters, global hemodynamic parameters [mean arterial pressure (MAP), heart rate (HR)] and cardiac output measured using echocardiography were recorded at 2 time points. In addition, several tissue perfusion parameters were collected at baseline and 1 h after vitamin C supplementation: arterial lactate level, index and knee capillary refill time and mottling score, skin temperature and central-skin temperature gradient.

### Plasma levels of vitamin C

Vitamin C plasma levels were measured by a high-performance liquid chromatography (HPLC) method adapted from Speek et al. [27]. Briefly, heparinized plasma is stabilized by diluting samples (1/10; v/v) with 5% (w/v) metaphosphoric acid solution. The samples remain frozen at − 80 °C until assayed. After alcalinization of samples with sodium acetate 4.5 mM (respectively 1 mL and 0.2 mL) and action of ascorbate oxidase (25 µL solution 62 U/mL in Na H_2_PO_4_ 4 mM pH 5.6; 5 min at 37 °C), the total vitamin C of the sample is converted into acid l-dehydroascorbate. This compound is derivatized with ortho-phenylenediamine (300 µL OPDA 100 mM-water solution, 30 min 37 °C) giving a fluorescent quinoxaline. The vitamin C assay is performed by HPLC in reverse phase with fluorimetric detection. The column is an Intersil C18 ODS2 5 µM 4.6 × 150 mm. The mobile phase (H2PO4 50 mM/methanol (500/214; v/v) pH 7.4) flow is 1.15 mL/min and the injection volume 20 µL. The excitation is done at 346 nm and emission at 424 nm. All the reagents are from Sigma-Aldrich, the column is from Interchim, and the HPLC system is a Summit Dionex-Thermo.

### Statistics

Continuous variables were presented as median and 25th–75th interquartile ranges (IQRs). Discrete variables were presented as percentages. Comparisons before and after vitamin C injection were made with a paired non-parametric test. Statistical analysis and graphical representations were performed using GraphPad Prism 8.4.1 software (Graph Pad Software Inc., La Jolla, CA). A two-sided *p* value of less than 0.05 was considered statistically significant.

### Ethics

The protocol was approved by an institution’s ethical committee—*Comité de Protection des Personne*s (*CPP Ile de France France*, 2019-A03199-48). All patients or their families gave their consent for the study (ClinicalTrials.gov Identifier: NCT04778605).

## Result

### Characteristics of included patients

During the study period, 30 septic shock patients were included. Median age was 67 [57–74] years with a higher proportion of men (70%). The main sources of infection were respiratory (43%) and abdominal (33%). Included patients had severe disease with high SOFA scores (11 [8–14]), high SAPS II (66 [54–79]) and frequent organ support therapy such as invasive mechanical ventilation (67%). In-ICU mortality was 33% (*N* = 10/30) (Table 1). Global hemodynamic and tissue perfusion parameters were measured after initial resuscitation. All patients received crystalloids (2.5 [2.1, 3.2] L) and norepinephrine to maintain MAP > 65 mmHg (dose 0.6 [0.3–1.2] µg/kg/min).

**Table 1:** General characteristics of included patients

Patients’ characteristics	*n* (%) or Med. [IQR]
Age	67 [57–74]
Body mass index (kg/m^2^)	22 [20-26]
Male gender	21 (70)
Simplified Acute Physiology Score 2	66 [54–79]
Sequential Organ Failure Assessment	11 [8–14]
*Comorbidities*	
Diabetes	7 (23)
Hypertension	14 (46)
Cardiovascular disease	10 (33)
Tobacco use	6 (20)
Cirrhosis	3 (10)
Cancer/hematologic malignancies	5 (17)
*Septic shock sources*	
Lung	13 (43)
Abdomen	10 (33)
Urinary tract	3 (10)
Catheter	2 (7)
Others	2 (7)
*Organ support therapy*	
Invasive mechanical ventilation	20 (67)
Norepinephrine dose (µg/kg/min)	0.6 [0.3–1.2]
Crystalloid infusion prior vitamin C (L)	2.50 [2.10–3.20]
Hydrocortisone	12 (40)
*Biological parameters at inclusion*	
Leucocytes (Giga/L)	11 (1.5–24)
Hemoglobin (g/dL)	10.6 (8.3–15.2)
Platelets (Giga/L)	132 (50–208)
Serum creatinine (µmol/L)	119 (83–182)
Procalcitonin (ng/mL)	12 (2.7–30)
Bicarbonate (mmol/L)	21 (17–24)
Arterial lactate (mmol/L)	3.9 (2.8–5.1)
Protidemia (g/L)	57 (46–63)
Serum albumin (g/L)	25 (22–31)
Vitamin C (µmol/L)	5.3 (2–17)

Biological parameters of included patients are depicted in Table 1.

### Microvascular blood flow parameters

**Fig. 1:**
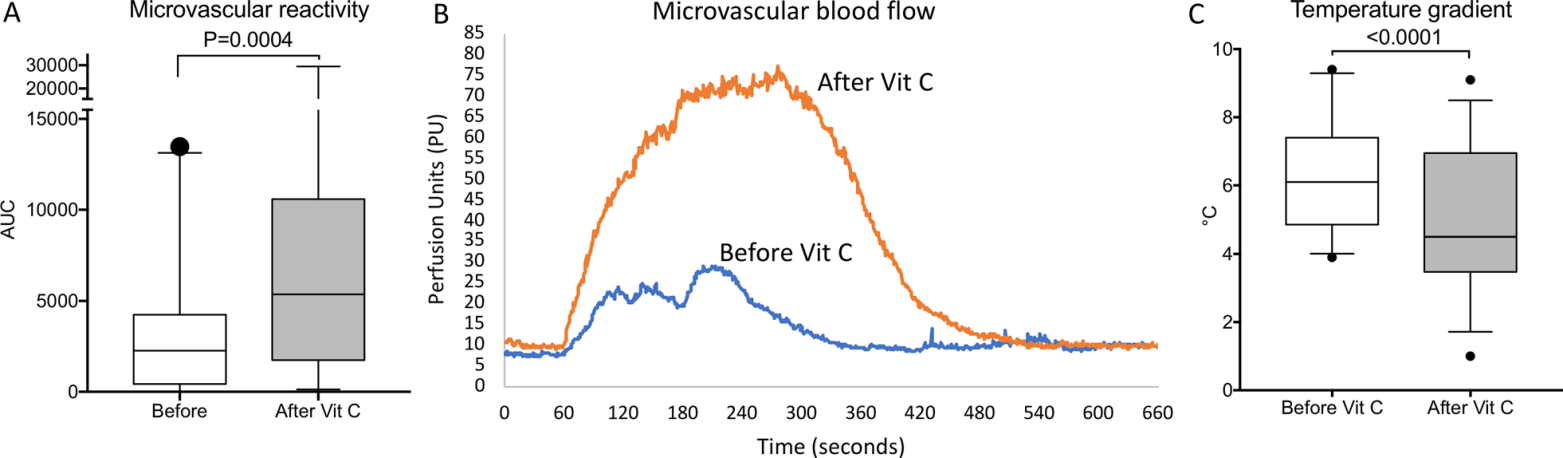
**A** Changes of forearm skin microcirculatory reactivity in response to acetylcholine challenge before and after vitamin C supplementation in patients with septic shock. **B** Example of skin microcirculatory blood flow change in response to acetylcholine iontophoresis before (blue) and after (orange) vitamin C injection. **C** Central-to-knee skin temperature gradient before and after vitamin C infusion. *PU* Perfusion units

Endothelial-dependent microvascular reactivity was measured in the forearm area after acetylcholine challenge before and 1 h after vitamin C administration. We observed that skin microvascular reactivity, evaluated using the area under the curve (AUC) during a 10-min monitoring period, strongly increased after vitamin C supplementation (AUC 2263 [430–4246] vs 5362 [1744–10585] UI, *p* = 0.0004) (Fig. 1A, B). Vitamin C improved microvascular reactivity in patients with and without peripheral tissue hypoperfusion (Additional file 2).

### Global hemodynamic and tissue perfusion parameters

**Fig. 2:**
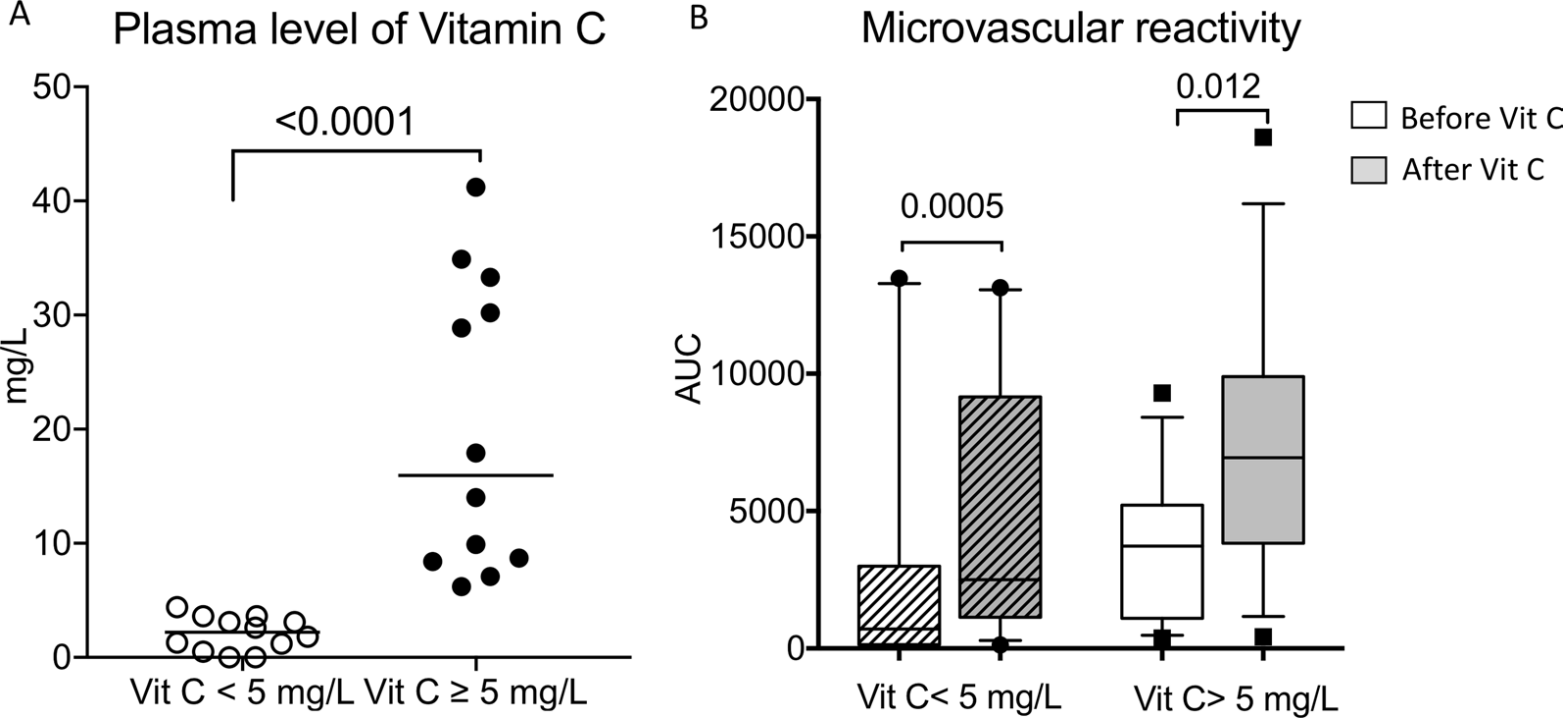
**A** plasma levels of vitamin C in patients with and without deficiency in patients with septic shock. **B** Changes of microcirculatory reactivity before and after vitamin C supplementation in septic shock patients with and without vitamin C deficiency

Parameters were recorded after initial resuscitation, before and 1 h after vitamin C supplementation. Following vitamin C infusion, cardiac output significantly decreased (4.1 (3.3–4.5) vs 4 (3.1–4.3) L/min, *p* = 0.0376) and MAP tended to increase (71 (66–75) vs 72 (66–77) mmHg; *p* = 0.07) despite no change in vasopressor dose (0.60 [0.30–1.10] vs 0.60 [0.30–1.20] µg/kg/min; *p* = 0.46) (Table 2). Interestingly, we observed that vitamin C supplementation quickly improved peripheral tissue perfusion with a trend to a decrease of mottling score (*p* = 0.06), and a significant decrease in finger-tip CRT (2.1 (1.7–3.5) vs 2 (1.2–3) s, *p* = 0.0003), Knee CRT (3.7 (2.6–5.5) vs 2.9 (1.9–4.7) s, *p* < 0.0001), skin temperature and central-to-skin temperature gradient (6.1 (4.9–7.4) vs 4.6 (3.4–7.0) °C, *p* < 0.0001) (Fig. 2C, Table 2).

**Table 2:** Global hemodynamic and tissue perfusion parameters before and 1 h after vitamin C infusion

Parameters, median (IQR)	H0	H1	*p* value
*Blood pressure (mmHg)*			
Systolic	107 (100–120)	111 (102–121)	0.43
Diastolic	54 (51–61)	58 (53–62)	0.18
Mean	71 (66–75)	72 (66–77)	0.07
Heart rate (/min)	107 (101–111)	107 (101–110)	0.67
Mottling score	1 (0–3)	1 (0–2)	0.06
*Capillary refill time (s)*			
Index	**2.1 (1.7–3.5)**	**2 (1.2–3)**	**0.0003**
Knee	**3.7 (2.6–5.5)**	**2.9 (1.9–4.7)**	**< 0.0001**
Cardiac output (L/min)	**4.1 (3.3–4.5)**	**4 (3.1–4.3)**	**0.0376**
Skin temperature	**31.2 (30.2–32.3)**	**32.2 (31–33.7)**	**< 0.0001**
Central-to-skin gradient temperature	**6.1 (4.9–7.4)**	**4.6 (3.4–7.0)**	**< 0.0001**
Norepinephrine dose (µg/kg min)	0.6 (0.3–1.2)	0.6 (0.3–1.1)	0.46

Bold was used when* p* value was ≤ 0.05

### Tissue perfusion parameters and microvascular parameters according to plasma levels of vitamin C

Plasma levels of vitamin C were measured in 24/30 septic shock patients at admission. Half of them (*N* = 12/24) had vitamin C deficiency (< 5 mg/L, [28]) (Fig. 2A). We did not observe any difference between no-deficiency and deficiency groups in terms of age, gender and co-morbidity, but time between hospital admission and ICU admission was longer in deficiency group patients (2 [1–5] vs 18 [2–39] days, *p* = 0.03). SOFA and SAPS II were not different between groups, but vasopressor doses trended to be lower in the vitamin C-deficient group (0.6 [0.2–0.7] µg/kg/min) vs 1.1 [0.4–1.4] µg/kg/min, *p* = 0.06) (Additional file 3).

We found that vitamin C supplementation significantly improved microvascular reactivity in patients with and without vitamin C deficiency (Fig. 2B), as well as bedside evaluated peripheral tissue perfusion (Table 3). Unexpectedly, we observed a significant positive correlation between baseline vitamin C levels and variations of endothelial response after supplementation (*r* = 0.64, *p* = 0.009); in other words, the higher the baseline vitamin C level, the higher the increase in blood flow after vitamin C supplementation (Additional file 4).

**Table 3:** Clinical and hemodynamic parameters at admission and 1 h after vitamin C infusion of included patients according to vitamin C deficiency

Variables, median (IQR)	H0		H1		*p* value (H1 vs H0)	
Vitamin C (µmol/L)	< 5 µmol/L	≥ 5 µmol/L	< 5 µmol/L	≥ 5 µmol/L	< 5 µmol/L	≥ 5 µmol/L
*Blood pressure (mmHg)*						
Systolic	103 [100–115]	108 [101–122]	109 [96–124]	110 [101–120]	0.26	0.8
Diastolic	56 [53–64]	53 [45–59]	58 [52–63]	56 [51–62]	0.9	0.11
Mean	71 [66–77]	**67 **[**61–71**]	74 [67–79]	**68 **[**64–75**]	0.29	**0.05**
Heart rate (/min)	105 [100–111]	107 [101–124]	105 [100–110]	107 [100–124]	0.8	0.9
Mottling score	1 [0–3]	2 [0–3]	1 [0–2]	2 [0–3]	0.6	0.9
*Capillary refill time (s)*						
Index	**1.8 **[**1.6–3.9**]	**2.2 **[**1.6–3.1**]	**1.7 **[**1.2–3.5**]	**1.9 **[**1.1–2.7**]	**0.04**	**0.02**
Knee	**3.8 **[**2.5–5.8**]	**4.1 **[**3.4–6**]	**2.7 **[**1.9–4.7**]	**3.5 **[**2.3–5.6**]	**0.001**	**0.005**
Cardiac output (L/min)	3.3 [3–4.2]	**4.3 **[**3.8–5.2**]	3.5 [3.1–4.2]	**4.3 **[**3–4.7**]	**0.5**	**0.04**
*Temperature (°C)*						
Skin	31.7 [30.2–32.7]	**31.2 **[**30.5–32.8**]	32 [31.1–34.3]	**32.2 **[**31.2–33.6**]	0.06	**0.008**
Central-to-skin gradient	6.5 [4.8–7.3]	**5.3 **[**4.7–6.8**]	4.9 [3.5–6.9]	**4.5 **[**3.3–6.6**]	0.06	**0.001**
Norepinephrine infusion (µg/kg min)	0.6 [0.2–0.7]	1.1 [0.4–1.4]	0.6 [0.2–0.7]	1.0 [0.4–1.4]	0.9	0.5

Bold was used when* p* value was ≤ 0.05

## Discussion

Our study prospectively investigated the impact of vitamin C infusion on microvascular function in septic shock patients. We found that vitamin C supplementation quickly improved microvascular reactivity and peripheral tissue perfusion, a benefit observed in patients with or without vitamin C deficiency.

Vitamin C supplementation was performed after initial resuscitation within the first 24 h of ICU admission. First, we observed that vitamin C trended to increase mean arterial pressure. Such effect may be due to the pleiotropic activity of vitamin C which promotes both the transformation of dopamine into norepinephrine and the endogenous catecholamine synthesis [29]. We found that vitamin C strongly increased skin microvascular blood flow after acetylcholine challenge, supporting an improvement in endothelial-dependent microvascular function. This finding is of great interest because microvascular reactivity is highly correlated with both septic shock severity and outcome: The lower the reactivity, the higher the mortality [30]. Acetylcholine specifically targets endothelial cells and promotes NO release, inducing vascular smooth muscle relaxation and *in fine* vasodilatation [22]. The beneficial effects of vitamin C on endothelial and nitric oxide dependent vasodilation have also been previously observed in patients with chronic endothelial dysfunction due to atherosclerosis [31], hypertension [31] or diabetes [32]. Such rapid effect observed one hour after vitamin C injection may be mediated by increased NO availability, either through enhanced synthesis mediated by BH4 recycling, direct reduction of nitrite to NO, release of NO from nitrosothiols, or by scavenging superoxide that would otherwise react with NO to form peroxynitrite [33]. Vitamin C may also limit BH4 oxidation, a key endothelial NOS cofactor [34]. Other protective effects of vitamin C on endothelial cell biology have been reported but these take longer time to develop. For instance, vitamin C promotes endothelial cell proliferation, capillary-like structures formation [35, 36] and prevents apoptosis both in vitro [37] and in vivo [38]. Vitamin C treatment limits Intercellular Adhesion Molecule (ICAM)-1 production by human umbilical endothelial cell line [39] and also decreases endothelial glycocalyx shedding in vivo, as assessed by plasma Syndecan-1 levels [40].

In our study, the beneficial effect of vitamin C supplementation was also observed clinically at the bedside with a decrease in mottling score, capillary refill time and temperature gradient, all markers of peripheral tissue perfusion. Mottling extension, which reflects impaired skin microvascular blood flow [41], has been identified as a strong independent predictive factor of mortality in sepsis [42] and septic shock patients [6]. Prolonged CRT measured either on the finger-tip or on the knee area is also associated with poor outcome in studies performed in the emergency ward [43] and the ICU [7]. Some criticisms have been raised about the reproducibility of these bedside parameters, but intra-rater concordance is excellent after standardization and training [44]. Skin temperature (and gradient) changes [8, 45], which were quantified with an accurate and reliable probe, also support the beneficial effect of vitamin C supplementation on peripheral perfusion.

In our cohort, around half of included patients had vitamin C deficiency, which is in line with previous works reporting that low plasma vitamin C concentrations are common in critically ill patients, and in particular in patients with sepsis [46, 47]. Vitamin C levels might be correlated with higher incidence of organ failure in septic patients [48], but in our study, SOFA score was not different patients with and without vitamin C deficiency. Several combined mechanisms may be responsible for vitamin C deficiency, such as pro-inflammatory cytokines regulating endothelial sodium-dependent vitamin C transporters activity [49] and increased vitamin C consumption by leukocyte turnover in the context of sepsis [50]. In our study, vitamin C-deficient group was characterized by longer in-hospital length of stay before ICU admission, with potential decreased vitamin C intake during hospital stay and also prolonged vitamin C consumption because of subacute sepsis. The beneficial effects of vitamin C were not restricted to vitamin C-deficient patients, since supplementation was also beneficial in septic shock patients without deficiency. The correlation between baseline vitamin C levels and variations of endothelial responses after supplementation was unexpected. Several potential explanations could be proposed: (1) We measured total vitamin C levels but not the oxidized and reduced forms which may have different vascular activity. (2) Recovery of impaired microvascular reactivity in Deficiency group after supplementation may require longer time and/or larger doses. (3) Some confounders between deficiency and no deficiency groups have not been identified.

Overall, the beneficial impact of vitamin C in sepsis patients is still into debate [51]. In a recent meta-analysis including eleven randomized controlled trials and more than 1700 patients, high-dose IV vitamin C did not improve short-term survival, but was associated with a significantly shorter duration of vasopressor use, as well as a significantly greater decline in the SOFA score at day 3 [52]. Based on our results, we believe that future trial testing high-dose IV vitamin C treatment should be proposed in selected septic shock patients with peripheral tissue hypoperfusion, a subset of patients with very poor outcome [53].

Finally, we did not observe any adverse effect after vitamin C injection, confirming previous work showing that pharmacologic ascorbic acid administration is safe. It is noteworthy that Sartor et al. reported that point-of-care blood glucose measurements may become inaccurate after ascorbate injection, since the molecular structures of vitamin C and glucose are somewhat similar [54]

Our study has several limitations. It is a monocentric study, and the results need to be confirmed in a larger population. Nevertheless, we found significative difference despite limited number of patients. Here, we did not include a control group and the improvement of peripheral tissue hypoperfusion could be, at least in part, related to initial resuscitation. However, rapid improvement of endothelial reactivity was unlikely due to fluid alone because in a previous work, we have shown in septic shock patients that saline infusion had no acute impact on endothelial dysfunction [55]. Vitamin C improved vascular parameters in septic shock patients under vasopressor support, but we cannot affirm that the protective effect would be still observed in sepsis patients without vasopressor. Finally, we observed beneficial effect of vitamin C early after infusion but we did not analyze microvascular function and peripheral tissue perfusion at later stages.

## Conclusion

In septic shock patients being resuscitated, vitamin C supplementation improved microvascular reactivity and peripheral tissue perfusion whatever plasma levels of vitamin C.

## Supplementary Information

**Additional file 1.** Figure showing typical recording of microvascular skin blood flow recorded by laser doppler flowmetry baseline and following 3 successive iontophoretic applications of Acetylcholine (Black arrows). AUC, area under curve, SBF, skin blood flow.

**Additional file 2.** Figure showing changes of microvascular reactivity according to baseline peripheral tissue perfusion in septic shock patients. Vitamin C administration improved microvascular perfusion in patients with (CRT > 2 s) and without (CRT > 2 s) impaired tissue perfusion. Data are expressed as median and IQRs. AUC for area under the curve and CRT for capillary refill time.

**Additional file 3.** Table describing general characteristics of patients according to plasma levels of vitamin C. SAPS 2, Simplified Acute Physiology Score 2, SOFA, Sequential Organ Failure Assessment.

**Additional file 4.** Correlations. A, Correlation between baseline plasma vitamin C levels and baseline Ach-induced microvascular blood flow. B, Correlation between baseline plasma vitamin C levels and variations of Ach-induced microvascular blood flow after supplementation. (Pearson’s correlation) Ach, Acetylcholine.

## Data Availability

Not applicable.

## Abbreviations

ACH: Acetylcholine
AUC: Area under the curve
CRT: Capillary refill time
ICU: Intensive care unit
MAP: Mean arterial pressure
SOFA: Sequential organ failure assessment
SAPS II: Simplified acute physiologic score II
