# Supplementary material for: Vitamin C improves microvascular reactivity and peripheral tissue perfusion in septic shock patients
Source: Crit Care. 2022 Jan 21;26:25. doi: 10.1186/s13054-022-03891-8 (PMC8781452; doi:10.1186/s13054-022-03891-8)
Supplement: Supplementary file 1 — Additional file 1. Figure showing typical recording of microvascular skin blood flow recorded by laser doppler flowmetry baseline and following 3 successive iontophoretic applications of Acetylcholine (Black arrows). AUC, area under curve, SBF, skin blood flow. [file 13054_2022_3891_MOESM1_ESM.pptx]

## Slide 1
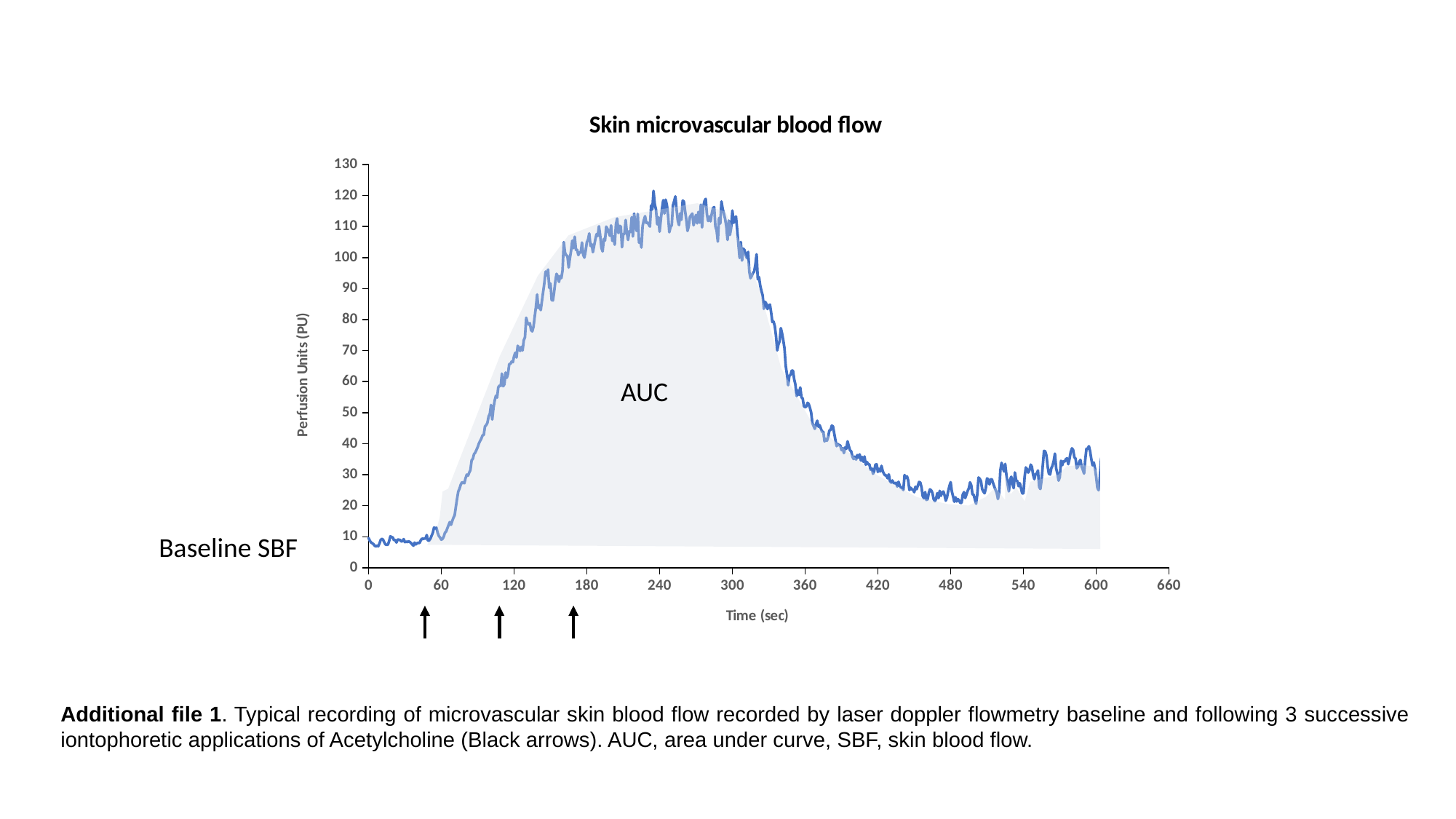

### Chart: Skin microvascular blood flow
| Category | |
|---|---|
AUC
Baseline SBF
Additional file 1. Typical recording of microvascular skin blood flow recorded by laser doppler flowmetry baseline and following 3 successive iontophoretic applications of Acetylcholine (Black arrows). AUC, area under curve, SBF, skin blood flow.
