# Supplementary material for: Vitamin C improves microvascular reactivity and peripheral tissue perfusion in septic shock patients
Source: Crit Care. 2022 Jan 21;26:25. doi: 10.1186/s13054-022-03891-8 (PMC8781452; doi:10.1186/s13054-022-03891-8)
Supplement: Supplementary file 2 — Additional file 2. Figure showing changes of microvascular reactivity according to baseline peripheral tissue perfusion in septic shock patients. Vitamin C administration improved microvascular perfusion in patients with (CRT > 2 s) and without (CRT > 2 s) impaired tissue perfusion. Data are expressed as median and IQRs. AUC for area under the curve and CRT for capillary refill time. [file 13054_2022_3891_MOESM2_ESM.pptx]

## Slide 1
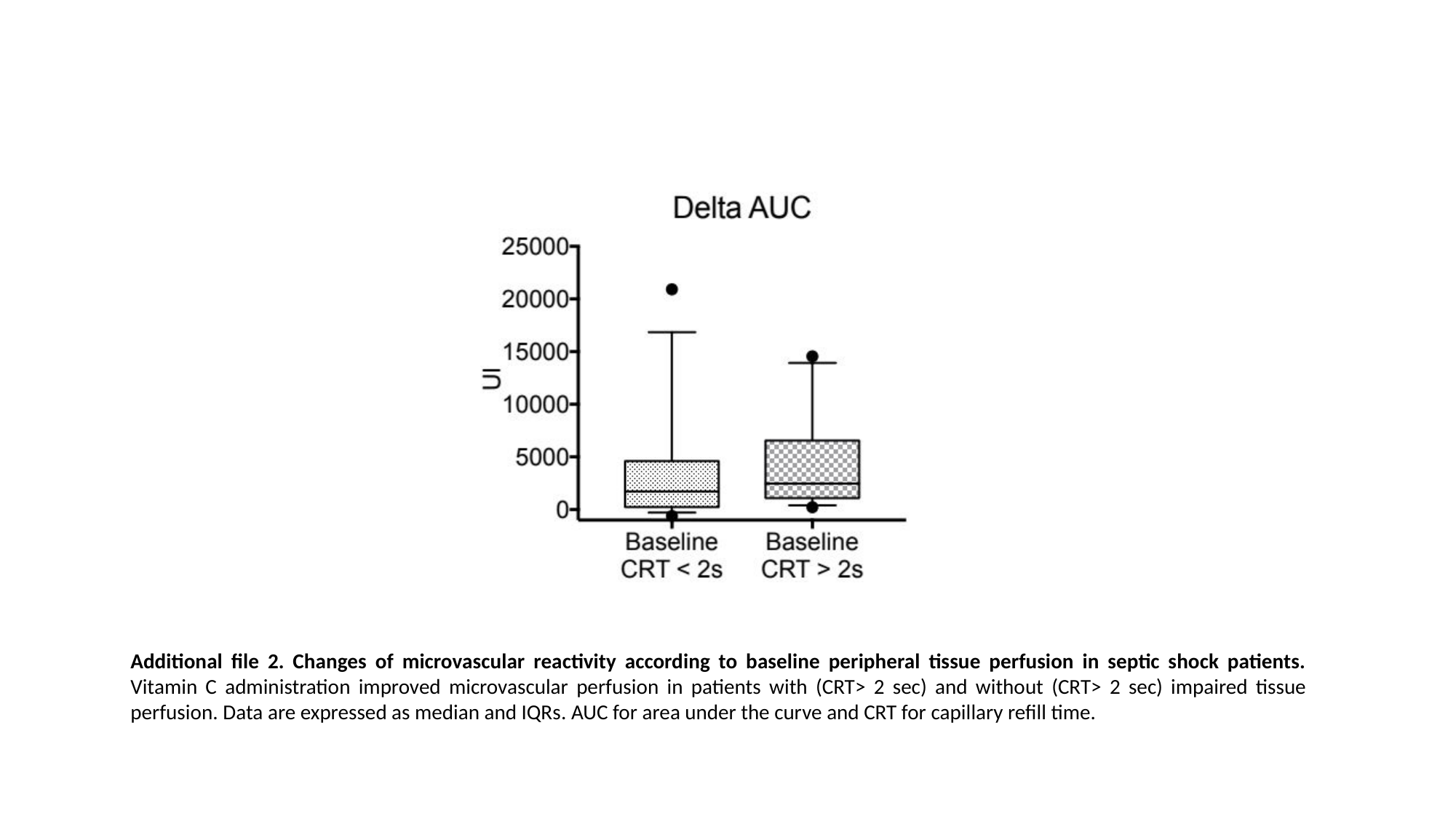

Additional file 2. Changes of microvascular reactivity according to baseline peripheral tissue perfusion in septic shock patients. Vitamin C administration improved microvascular perfusion in patients with (CRT> 2 sec) and without (CRT> 2 sec) impaired tissue perfusion. Data are expressed as median and IQRs. AUC for area under the curve and CRT for capillary refill time.
