# Supplementary material for: Vitamin C improves microvascular reactivity and peripheral tissue perfusion in septic shock patients
Source: Crit Care. 2022 Jan 21;26:25. doi: 10.1186/s13054-022-03891-8 (PMC8781452; doi:10.1186/s13054-022-03891-8)
Supplement: Supplementary file 3 — Additional file 3. Table describing general characteristics of patients according to plasma levels of vitamin C. SAPS 2, Simplified Acute Physiology Score 2, SOFA, Sequential Organ Failure Assessment. [file 13054_2022_3891_MOESM3_ESM.pptx]

## Slide 1
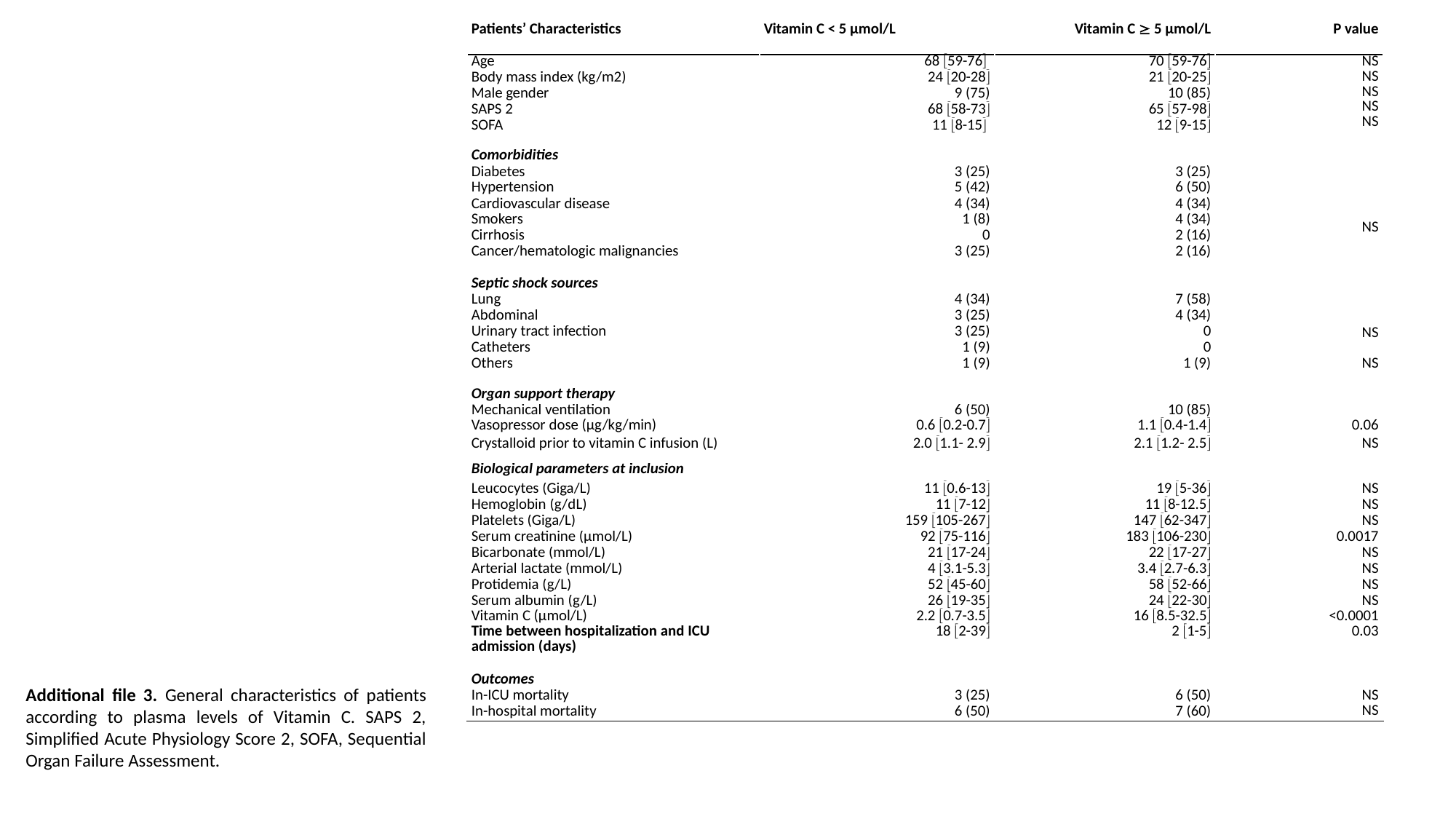

| Patients’ Characteristics | Vitamin C < 5 µmol/L | Vitamin C ≥ 5 µmol/L | P value |
| --- | --- | --- | --- |
| Age | 68 59-76 | 70 59-76 | NS NS NS NS NS             NS             NS   NS |
| Body mass index (kg/m2) | 24 20-28 | 21 20-25 | |
| Male gender | 9 (75) | 10 (85) | |
| SAPS 2 | 68 58-73 | 65 57-98 | |
| SOFA | 11 8-15 | 12 9-15 | |
| Comorbidities | | | |
| Diabetes | 3 (25) | 3 (25) | |
| Hypertension | 5 (42) | 6 (50) | |
| Cardiovascular disease | 4 (34) | 4 (34) | |
| Smokers | 1 (8) | 4 (34) | |
| Cirrhosis | 0 | 2 (16) | |
| Cancer/hematologic malignancies | 3 (25) | 2 (16) | |
| Septic shock sources | | | |
| Lung | 4 (34) | 7 (58) | |
| Abdominal | 3 (25) | 4 (34) | |
| Urinary tract infection | 3 (25) | 0 | |
| Catheters | 1 (9) | 0 | |
| Others | 1 (9) | 1 (9) | |
| Organ support therapy | | | |
| Mechanical ventilation | 6 (50) | 10 (85) | |
| Vasopressor dose (µg/kg/min) | 0.6 0.2-0.7 | 1.1 0.4-1.4 | 0.06 |
| Crystalloid prior to vitamin C infusion (L) | 2.0 1.1- 2.9 | 2.1 1.2- 2.5 | NS |
| Biological parameters at inclusion | | | |
| Leucocytes (Giga/L) | 11 0.6-13 | 19 5-36 | NS |
| Hemoglobin (g/dL) | 11 7-12 | 11 8-12.5 | NS |
| Platelets (Giga/L) | 159 105-267 | 147 62-347 | NS |
| Serum creatinine (µmol/L) | 92 75-116 | 183 106-230 | 0.0017 |
| Bicarbonate (mmol/L) | 21 17-24 | 22 17-27 | NS |
| Arterial lactate (mmol/L) | 4 3.1-5.3 | 3.4 2.7-6.3 | NS |
| Protidemia (g/L) | 52 45-60 | 58 52-66 | NS |
| Serum albumin (g/L) | 26 19-35 | 24 22-30 | NS |
| Vitamin C (µmol/L) | 2.2 0.7-3.5 | 16 8.5-32.5 | <0.0001 |
| Time between hospitalization and ICU admission (days) | 18 2-39 | 2 1-5 | 0.03 |
| Outcomes | | | |
| In-ICU mortality | 3 (25) | 6 (50) | NS NS |
| In-hospital mortality | 6 (50) | 7 (60) | |
Additional file 3. General characteristics of patients according to plasma levels of Vitamin C. SAPS 2, Simplified Acute Physiology Score 2, SOFA, Sequential Organ Failure Assessment.
