# Supplementary material for: Vitamin C improves microvascular reactivity and peripheral tissue perfusion in septic shock patients
Source: Crit Care. 2022 Jan 21;26:25. doi: 10.1186/s13054-022-03891-8 (PMC8781452; doi:10.1186/s13054-022-03891-8)
Supplement: Supplementary file 4 — Additional file 4. Correlations. A, Correlation between baseline plasma vitamin C levels and baseline Ach-induced microvascular blood flow. B, Correlation between baseline plasma vitamin C levels and variations of Ach-induced microvascular blood flow after supplementation. (Pearson’s correlation) Ach, Acetylcholine. [file 13054_2022_3891_MOESM4_ESM.pptx]

## Slide 1
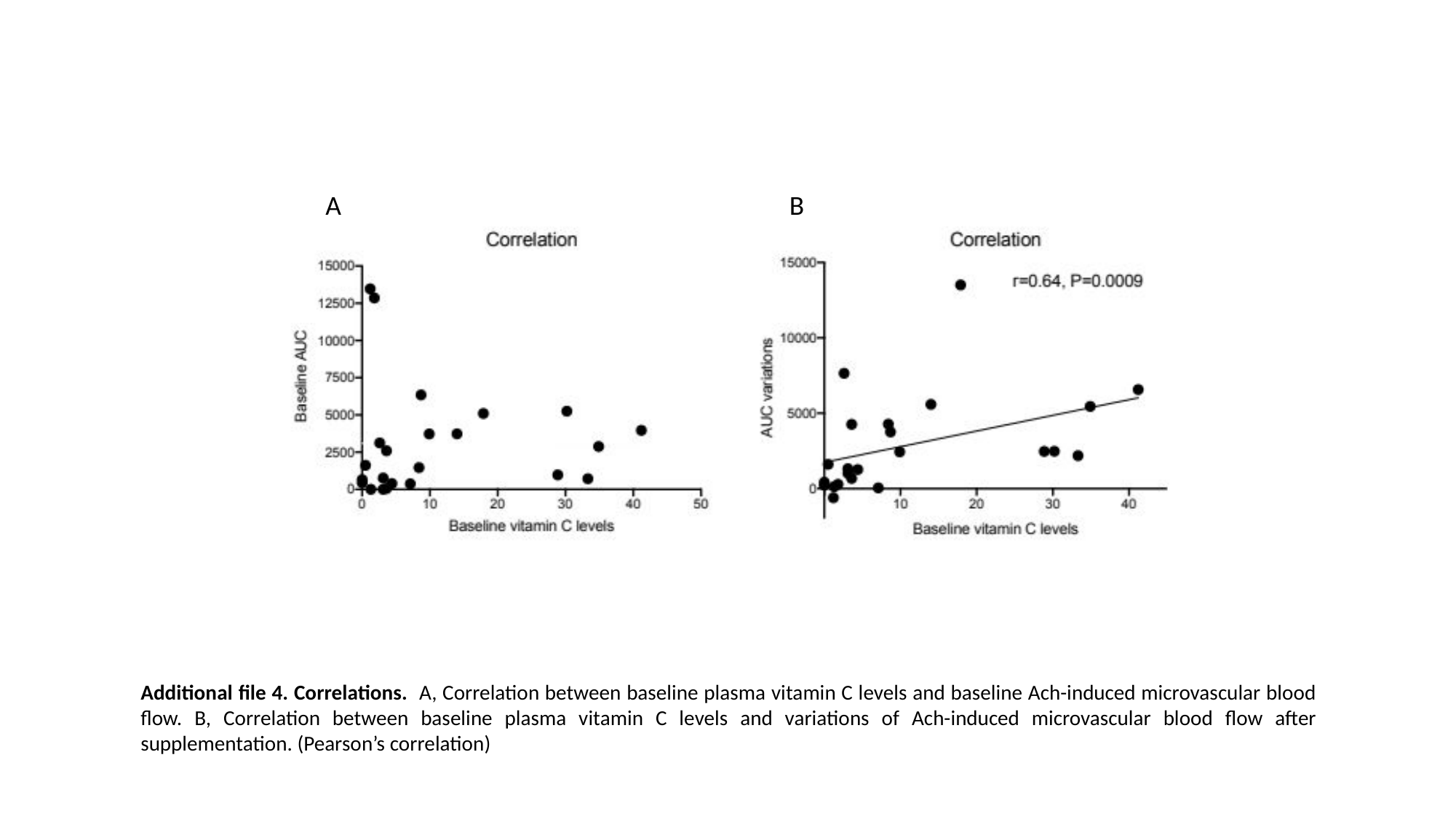

A
B
Additional file 4. Correlations. A, Correlation between baseline plasma vitamin C levels and baseline Ach-induced microvascular blood flow. B, Correlation between baseline plasma vitamin C levels and variations of Ach-induced microvascular blood flow after supplementation. (Pearson’s correlation)
